# Draft Genome Sequence of *Chromobacterium pseudoviolaceum* LMG 3953^T^, an Enigmatic Member of the Genus *Chromobacterium*

**DOI:** 10.1128/genomeA.01632-16

**Published:** 2017-03-23

**Authors:** Scott D. Soby

**Affiliations:** Biomedical Sciences Program, College of Health Sciences and College of Veterinary Medicine, Midwestern University, Glendale, Arizona, USA

## Abstract

*Chromobacterium pseudoviolaceum* LMG 3953^T^ was separated from *Chromobacterium violaceum* in 2009, but little is known of its origin or environmental role. Here, the genome of LMG 3953^T^ was sequenced to understand the evolution of the genus *Chromobacterium*. It is not clear from this sequence that *C. pseudoviolaceum* is taxonomically distinct from *C. violaceum*.

## GENOME ANNOUNCEMENT

*Chromobacterium pseudoviolaceum* LMG 3953^T^ is an isolate of uncertain origin that was originally classified as *Chromobacterium violaceum* (1), but it was redefined and renamed in 2009 as part of a recent expansion of the genus (2). The environmental or functional status of *C. pseudoviolaceum* is unclear, and there is uncertainty about the taxonomic status of the genus (3–6). The completion of a collection of genomic sequences of all of the species with standing in the literature will be important in redefining the genus. The genome of *C. pseudoviolaceum* LMG 3953^T^ was sequenced at the Arizona State University CLAS Genomics Core facility using Illumina MiSeq. Genomic DNA was sheared to approximately 600-bp fragments using a Covaris M220 ultrasonicator, and Illumina libraries were generated on an Apollo 384 liquid handler (Wafergen) using a Kapa Biosystems library preparation kit (catalog no. KK8201). DNA fragments were end-repaired and A-tailed as described in the Kapa protocol. Combined indexes/adapters (catalog no. 520999; Bioo) were ligated onto each sample and multiplexed into one lane. Adapter-ligated molecules were cleaned using AMPure beads (catalog no. A63883; Agencourt Bioscience/Beckman Coulter, Inc.) and amplified with Kapa HIFI enzyme. Libraries were analyzed on an Agilent Bioanalyzer and quantified by quantitative PCR (qPCR) (catalog no. KK4835; Kapa library quantification kit) before multiplex pooling and sequencing in a 2 × 300 paired-end (PE) flow cell on the MiSeq platform (Illumina). Adapters were computationally segregated and trimmed in the Illumina BaseSpace pipeline. The Velvet assembly tool (BaseSpace) was used for signal processing and partial sequence assembly. The sequence is 64.72% G+C and consists of 4,660,272 bp distributed over 480 scaffolds, 220 of which are larger than 1 kbp. The largest contig is 226,360 bp, the *N*_50_ is 44,500 bp, and the *N*_75_ is 23,442 bp, with a sequence coverage of 50.37×.

*Ab initio* gene prediction was performed on the assembly using RAST (http://rast.nmpdr.org/). There are 4,286 predicted genes in the genome, of which about half are identifiable in the RAST/SEED servers. Like many of the other *Chromobacterium* spp., the *C. pseudoviolaceum* LMG 3953^T^ genome contains homologs to *Mycobacterium* virulence operons for protein synthesis, DNA transcription, quinolinate and fatty acid biosynthesis, as well as chitinase and *N*-acetylglucosamine transport pathways. Genes are present for the synthesis of enterobactin siderophores, cyanate hydrolysis, lysozyme inhibitors, and heme/hemin uptake systems. Each of these pathways can be related to virulence, although there is no report of *C. pseudoviolaceum* causing disease. Surprisingly, and unlike other members of the genus, there are no detectable transposon-related genes present. The *Chromobacterium pseudoviolaceum* LMG 3953^T^ genome sequence was compared to reference genomes of *C. violaceum* ATCC 12472, *Chromobacterium haemolyticum*, *Chromobacterium vaccinii*, *Chromobacterium piscinae*, *Chromobacterium aquaticum*, *Chromobacterium* sp. strains LK1, LK11, and 49, and *Chromobacterium subtsugae* using the Genome-to-Genome Distance Calculator (GGDC) (7, 8). The *C. pseudoviolaceum* LMG 3953^T^ genome is less than 41% homologous to these reference genomes, except to *C. violaceum* ATCC 12472, which was 84.4% homologous, calling into question the separation of *C. pseudoviolaceum* from *C. violaceum*.

### Accession number(s).

This whole-genome shotgun project has been deposited at DDBJ/EMBL/GenBank under the accession number MQZX00000000. The version described in this paper is version MQZX01000000.
